# Biocompatible PEO-b-PCL Nanosized Micelles as Drug Carriers: Structure and Drug–Polymer Interactions

**DOI:** 10.3390/nano10091872

**Published:** 2020-09-18

**Authors:** Angeliki Chroni, Thomas Mavromoustakos, Stergios Pispas

**Affiliations:** 1 Theoretical and Physical Chemistry Institute, National Hellenic Research Foundation, 48 Vassileos Constantinou Avenue, 11635 Athens, Greece; angelikechrone@gmail.com; 2 Department of Chemistry, National and Kapodistrian University of Athens, Panepistimioupolis, 15771 Zografou, Greece; tmavrom@chem.uoa.gr

**Keywords:** amphiphilic block copolymers, polymeric nanocarriers, drug delivery systems, organic solvent evaporation method, drug encapsulation, drug–polymer intermolecular interactions

## Abstract

We report on the preparation of drug nanocarriers by encapsulating losartan potassium (LSR) into amphiphilic block copolymer micelles, utilizing the biocompatible/biodegradable poly(ethylene oxide)-b-poly(ε-caprolactone) (PEO-b-PCL) diblock copolymer. The PEO-b-PCL micelles and LSR-loaded PEO-b-PCL nanocarriers were prepared by organic solvent evaporation method (OSEM). Light scattering and nuclear magnetic resonance (NMR) provide information on micelle structure and polymer–drug interactions. According to dynamic light scattering (DLS) analysis, the PEO-b-PCL micelles and LSR-loaded PEO-b-PCL nanocarriers formed nanostructures in the range of 17–26 nm in aqueous milieu. Attenuated total reflection Fourier transform infrared (ATR-FTIR) and ultraviolet-visible (UV-Vis) measurements confirmed the presence of LSR in the polymeric drug solutions. NMR results proved the successful encapsulation of LSR into the PEO-b-PCL micelles by analyzing the drug–micelles intermolecular interactions. Specifically, 2D-NOESY experiments clearly evidenced the intermolecular interactions between the biphenyl ring and butyl chain of LSR structure with the methylene signals of PCL. Additionally, NMR studies as a function of temperature demonstrated an unexpected, enhanced proton mobility of the PEO-b-PCL micellar core in D_2_O solutions, probably caused by the melting of the PCL hydrophobic core.

## 1. Introduction

Significant research focus in the past few decades has shifted towards the design of drug-loaded block copolymer nanocarriers for addressing and treating challenging diseases [1,2,3,4,5]. Nano-drug delivery systems based on amphiphilic block copolymers (AmBCs) such as micelles, liposomes, dendrimers, etc., have been extensively studied since they are meeting requirements for enhanced stability of the micellar assembly, drug solubility, prolonged circulation times, and controlled release of the drug [6,7,8,9]. 

Block copolymer micelles are a result of a spontaneous and intriguing self-assembly of AmBCs in aqueous milieu [10]. Particularly, block copolymer micelles are supramolecular, self-assembled, and well-defined nano-scale colloidal structures composed of a hydrophobic core that can physically encapsulate drug molecules enhancing their solubility and reducing their toxicity, surrounded by a hydrophilic shell that prolongs circulation time in blood, stabilizes the core, and increases accumulation in tumor tissues [11,12,13]. 

The poly(ethylene oxide)-b-poly(ε-caprolactone) (PEO-b-PCL) block copolymer system has been the focus of extensive research in the field of drug delivery over the past decade, unveiling its high biocompatibility, biodegradability, and low toxicity [14]. PCL is recognized as a semi-crystalline and biodegradable polymer that contributes to the solubilization of lipophilic drugs and forms the hydrophobic core of the polymeric micelles. The biocompatible PEO serves as the hydrophilic shell providing stealth properties to the micellar system, preventing its uptake by the reticuloendothelial system (RES) [15]. To date, several studies have reported the development of PEO-b-PCL polymeric micelles as effective vehicles for the solubilization and delivery of doxorubicin [16], paclitaxel [17], curcumin [18,19], indomethacin [20], camptothecin [21], tacrolimus [22], cyclosporine [23], amiodarone [24], hydrocortisone [25], clotrimazole [15], cucurbitacin [26], among others.

Sartans is a class of new antihypertensive drugs, which compete with the hypertensive action of the vasoconstrictor hormone Angiotensin II (ANG II) at the ANG II type 1 (AT_1_) receptor site, modulating the renin-angiotensin–aldosterone system (RAS) [27]. Losartan potassium (LSR) is an ANG II receptor blocker and is generally used in the treatment of hypertension [28,29,30]. Nevertheless, recent studies have demonstrated that the encapsulation of LSR both in liposomes and peptide hydrogels improves the effectiveness of chemotherapy, thanks to collagen network inhibition that greatly prevents nanotherapeutics from penetrating the tumor [31,32]. Lately, LSR has proved beneficial for the treatment of breast cancer [33] and depression [34]. In another study, hyaluronic acid (HA) micelles carrying LSR suggested as alternative vehicles for treating liver fibrosis [35]. However, AT_1_ antagonists suffer from lipophilicity and block copolymer nanocarriers are needed to increase their bioavailability.

The degree of compatibility or interaction between the polymer and the drug is expected to influence many important features, including stability, encapsulation efficiency, and drug release kinetics. Nuclear magnetic resonance (NMR) spectroscopy is widely viewed as the most powerful tool for structural elucidation of organic compounds but also for detecting intra- and intermolecular interactions of biomolecules [36,37] and Pluronics systems [38,39].

To the best of our knowledge, the use of NMR spectroscopy in the analysis of the drug–polymer intermolecular interactions and the drug encapsulation into the micelles has been scarcely explored in the literature. 

Here, we developed a drug delivery system by encapsulating LSR into a biocompatible PEO-b-PCL block copolymer and examined the intramolecular and intermolecular interactions between the polymer and the drug using NMR spectroscopy. The PEO-b-PCL micelles and LSR-loaded PEO-b-PCL nanocarriers (20 wt. % and 50 wt. % concentration of LSR in the mixture) were prepared using the organic solvent evaporation method (OSEM). An extensive physicochemical characterization was conducted using dynamic and electrophoretic light scattering (DLS, ELS) and attenuated total reflection Fourier transform infrared (ATR-FTIR) spectroscopy to assess the size, the surface potential, and the structure both of the PEO-b-PCL micelles and LSR-loaded PEO-b-PCL nanocarriers (referred in the text as PEO-b-PCL+20% LSR and PEO-b-PCL+50% LSR nanocarriers). The stability of the resulting nanocarriers was studied over a certain period of time using DLS. The encapsulation of LSR into PEO-b-PCL micelles was examined through ultraviolet-visible (UV-Vis), ATR-FTIR, and NMR measurements in aqueous solutions. Particularly, proton–NMR (^1^H-NMR) experiments yielded insight into the internal structure and the aggregation behavior of the micelles and verified the presence of LSR in the polymer/drug nanocarriers. Experiments on correlated spectroscopy (2D COSY) were further performed to analyze and detect the intramolecular interactions between the polymer and the drug. In addition, the Overhauser effect (2D NOESY) and diffusion-ordered (2D DOSY) spectroscopies were applied to explore the drug–micelles intermolecular interactions and determine the self-diffusion coefficients D of the nanocarriers. At last, temperature studies using ^1^H-NMR spectroscopy for the PEO hydrophilic homopolymer and the PEO-b-PCL copolymer over the temperature range 25–80 °C were pursued to trace the mobility of protons located in the hydrophobic micellar core.

## 2. Materials and Methods

### 2.1. Materials

Losartan potassium (LSR, 99.8%, Rafarm, Athens, Greece), tetrahydrofuran (THF, 99.9%, Sigma-Alrdrich, Athens, Greece), water for injection (WFI, 99%, Sigma-Alrdrich), deuterium oxide (D_2_O, 99%, Sigma-Alrdrich). The synthesis of PEO-b-PCL copolymer was performed using the monohydroxy terminated PEO (M_n_ = 5000, Sigma-Alrdrich) as the macroinitiator for ε-caprolactone (CL) (Sigma-Alrdrich) polymerization using stannous octoate (Sigma-Alrdrich) as the catalyst.

### 2.2. PEO-b-PCL Block Copolymer Synthesis

**Scheme 1:**
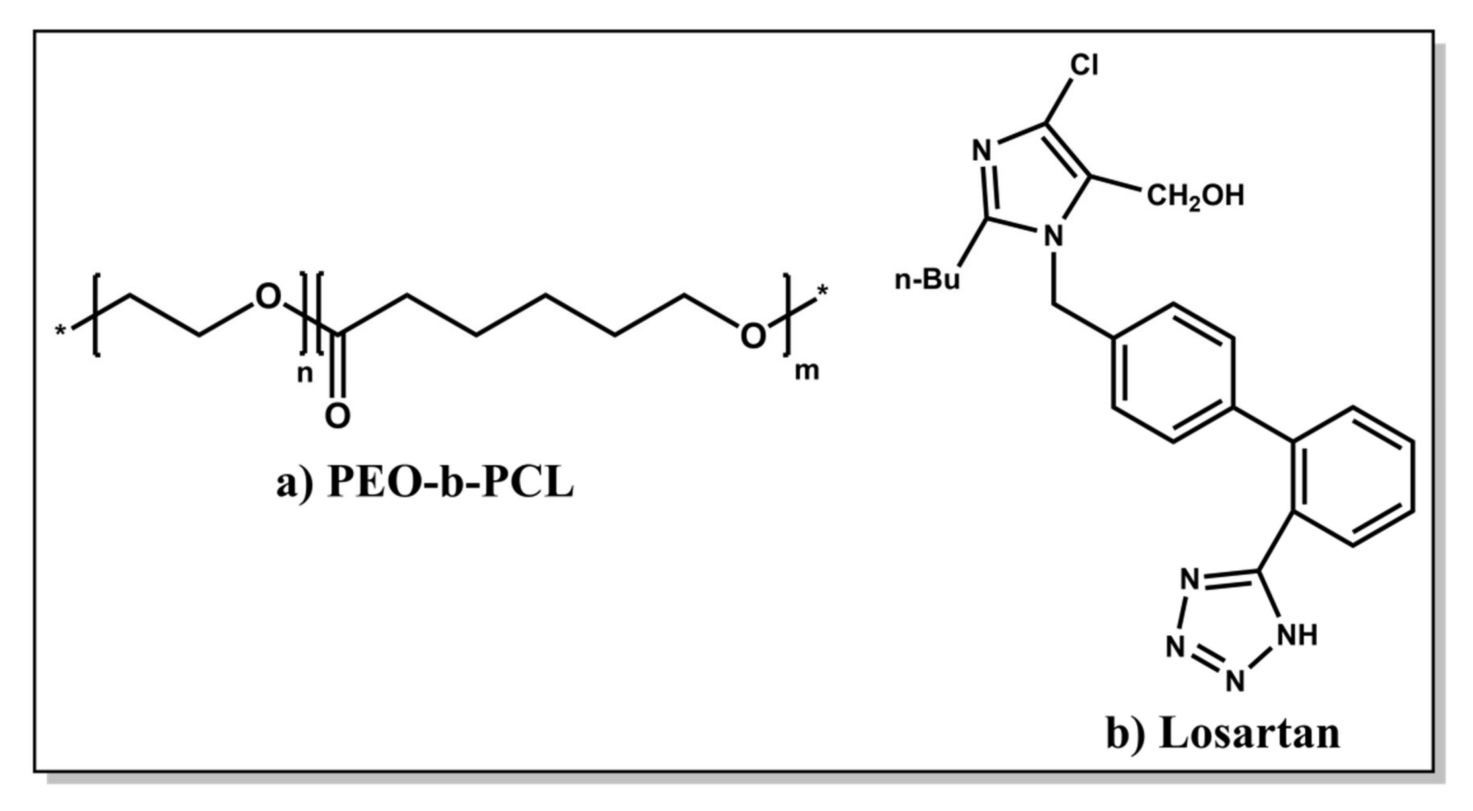
Chemical structures of (**a**) PEO-b-PCL diblock copolymer synthesized by ROP of CL and (**b**) LSR. Asterisk in the chemical structure of the copolymer denotes the presence of end groups at the ends of the copolymer chain.

The synthesis of PEO-b-PCL block copolymer was performed by ring opening polymerization (ROP) of CL monomer, and it has been described in detail in a previous work [20]. The molecular characteristics of the PEO-b-PCL copolymer and the chemical structures both of the copolymer and LSR are provided in Table 1 and Scheme 1, respectively.

### 2.3. Self-Assembly of PEO-b-PCL Block Copolymer

Due to the amphiphilic character of the PEO-b-PCL diblock, its solution behavior was studied in aqueous solutions at a polymer concentration of C = 10^−3^ g/mL using the OSEM. Specifically, 5 mg of the PEO-b-PCL diblock are firstly dissolved in THF (stock solution) and allowed to stand overnight for polymer dissolution at the molecular level. After 24 h, 5 mL of water for injection are added in a vial with a magnetic stirrer placed over a stirring plate (600 rpm) for the fast injection of the THF PEO-b-PCL stock solution in the stirred water. Afterwards, the final PEO-b-PCL solution is placed into a warm water bath (40 °C) on a hot stirring plate for approximately 30 min until the efficient and gentle evaporation of THF.

### 2.4. Preparation of LSR-Loaded PEO-b-PCL Nanocarriers

The LSR-loaded PEO-b-PCL nanocarriers were also prepared using the OSEM. Particularly, an appropriate amount of LSR is dissolved in THF to prepare 20 wt. % and 50 wt. % concentration of LSR in the final copolymer/drug mixture. After 24 h, the copolymer and LSR stock solutions are mixed in the appropriate amounts. Then, 5 mL of water for injection are added in a vial with a magnetic stirrer placed over a stirring plate (600 rpm) for the fast injection of PEO-b-PCL/LSR THF solution in the stirred water. Finally, the final PEO-b-PCL/LSR solution is placed into a warm water bath (40 °C) on a hot stirring plate for approximately 30 min until the efficient and gentle evaporation of THF. All copolymer and drug-loaded solutions were filtered through 0.45 μm pore size filters and allowed to stand overnight for equilibration before measurements.

### 2.5. NMR Samples Preparation

Temperature studies of PEO homopolymer and PEO-b-PCL copolymer: 2 mg of PEO/PEO-b-PCL diblock were dissolved to 0.7 mL D_2_O. 

PEO-b-PCL micelles and LSR-loaded PEO-b-PCL nanocarriers were prepared by OSEM in D_2_O solutions as follows: 1 mg of PEO-b-PCL diblock was dissolved to 1 mL D_2_O. The partitioning of the drug in the PEO-b-PCL micelles was studied by adding an appropriate amount of LSR (to prepare 50 wt. % concentration of LSR) in the final copolymer/drug mixture. 

### 2.6. Methods

^1^H-NMR spectroscopy studies of PEO-b-PCL micelles and LSR-loaded PEO-b-PCL nanocarriers were carried out on 600 MHz NMR spectrometer (Agilent Technologies, Palo Alto, CA, USA) operated by Vjnmr software and with a 5 mm HCN cold probe. Tetramethylsilane (TMS) was the internal standard used and D_2_O as the solvent. ^1^H-NMR spectra were recorded with 65,536 points, 90° pulse, 10 s relaxation delay, and 32 repetitions. 2D-COSY, NOESY, and DOSY were reported on 600 MHz NMR spectrometer. NOESY spectra were recorded at different mixing times with 4096 × 200 points, 1 s relaxation delay and 32 repetitions per spectrum. The DgcsteSL_cc sequence was used to record DOSY spectra with 65,536 points, 1 s relaxation delay and 16 repetitions. Twenty-four gradient strengths between zero and 60 gauss/cm were used. All spectra were recorded at 25 °C. Chemical shifts are referenced with respect to the lock frequency and reported relative to TMS. Temperature studies of PEO homopolymer and PEO-b-PCL copolymer were performed on a Varian 300 MHz NMR spectrometer (Agilent Technologies, Palo Alto, CA, USA). The composition of PEO-b-PCL diblock copolymer was reported in a previous work [20].

DLS measurements were performed using an ALV/CGS-3 Compact Goniometer System (ALV GmbH, Siemensstraße 4, 63,225 Langen (Hessen, Germany) with an ALV-5000/EPP multi-tau digital correlator of 288 channels and an ALV/LSE-5003 light scattering electronic unit for stepper motor drive and limit switch control. A JDS Uniphase 22-mW He-Ne laser (632.8 nm) was used as the light source. The size data and figures provided in the manuscript are from averaged measurements at 90 degrees, (five measurements per concentration/angle). The obtained correlation functions were analyzed by the cumulants method and CONTIN software (ALVGmbH, Hessen, Germany). All solutions were filtered through 0.45 μm hydrophilic PTFE filters (Millex-LCR from Millipore, Billerica, MA, USA) before measurements.

ZetaSizer Nanoseries Nano-ZS (Malvern Instruments Ltd., Malvern, UK) was used for the ELS measurements, equipped with a 4-mW solid-state laser at a wavelength of 633 nm and a fixed backscattering angle of 173°. The reported zeta-potential (ζ_pot_) values are the average values of 50 runs, utilizing the Henry approximation of Smoluchowski equation after equilibration at 25 °C.

UV–Vis absorption spectra of LSR and LSR-loaded PEO-b-PCL nanocarriers were recorded between 200 and 600 nm wavelength using a Perkin Elmer (Lambda 19) UV–Vis–NIR spectrophotometer (Waltham, MA, USA). The LSR-loaded PEO-b-PCL nanocarriers were diluted to get an absorbance value of less than 1. It is notable that the absorption at 260 nm is related to the presence of LSR and not of the copolymer based on measurements on the pure components.

Mid-infrared (IR) spectra of LSR, PEO-b-PCL micelles, and LSR-loaded PEO-b-PCL nanocarriers were recorded in the region 550–4000 cm^−1^ on an Equinox 55 FTIR spectrometer (Bruker, Billerica, MA, USA) equipped with a single reflection diamond ATR accessory (DuraSamp1IR II by SensIR Technologies).

ATR-FTIR spectral peaks of PEO-b-PCL micelles: v (cm^−1^) = 2700–3050 (–CH–), 1724 (–C=O–), 1464 and 1358 (–CH_2_–), 1240 and 1189 (–C–O–C–), 1108 (–CO–), 841 (Crystalline PEO), 734 (Crystalline PCL).

## 3. Results and Discussion

### 3.1. Synthesis and Molecular Characterization of PEO-b-PCL Diblock Copolymer

The synthetic procedure and molecular characterization of the amphiphilic PEO-b-PCL diblock copolymer is presented and discussed in a previous work [20]. The molecular characteristics are shown in Table 1 and the chemical structures of the copolymer and LSR are presented in Scheme 1.

### 3.2. Physicochemical Characterization of the PEO-b-PCL Micelles

**Figure 1:**
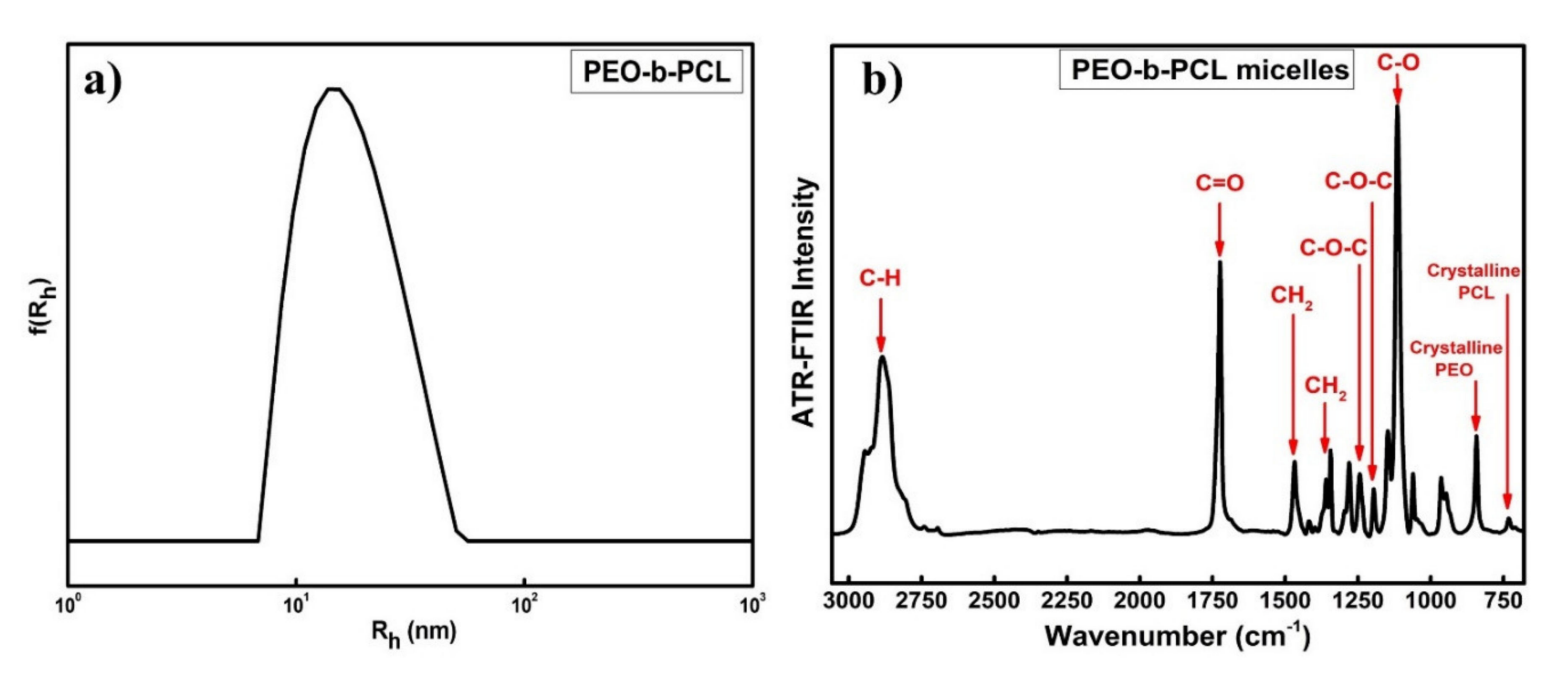
(**a**) Size distribution from Contin for the PEO-b-PCL diblock at 90° in aqueous solutions using the organic solvent evaporation method, (**b**) attenuated total reflection Fourier transform (ATR-FTIR) spectra of PEO-b-PCL micelles in aqueous solutions using the OSEM.

An extensive physicochemical characterization is provided for the PEO-b-PCL micelles using DLS, ELS, and ATR-FTIR techniques. According to existing literature, PEO-b-PCL copolymer is expected to self-assemble into micelles when inserted in aqueous solutions, where a PCL block forms the hydrophobic core and PEO the hydrophilic corona [16,20,23,24,40,41,42,43]. As seen from the size distribution plot in Figure 1a, DLS data confirm this scenario. The intensity weighted and monomodal size distribution plot from Contin analysis in Figure 1a reveals the existence of one symmetrical peak, indicating the participation of all chains in the formation of block copolymer micelles (R_h_ = 17 nm). The hydrodynamic radius (R_h_) and polydispersity index (PDI) values of the PEO-b-PCL copolymer formed in aqueous media at C=10^−3^ g/mL, pH=7, and 25 °C are summarized in Table 2.

ELS measurements of the PEO-b-PCL copolymer in aqueous media revealed a slightly negative surface potential, ζ_pot_ = −6 mV (Table 2), falling within the range of values usually reported for PEO-b-PCL block copolymer micelles [20].

ATR-FTIR measurements can certify the chemical structure of the PEO-b-PCL micelles. Figure 1b exhibits the ATR-FTIR spectra of PEO-b-PCL micelles in aqueous media. The FTIR spectrum of the PEO-b-PCL micelles presents the characteristic absorption bands due to the stretching vibrations of aliphatic C–H at 3050–2700 cm^−1^ region, carbonyl C=O stretching vibration at 1724 cm^−1^, −CH_2_ bending vibrations at 1464 and 1358 cm^−1^, asymmetric and symmetric stretching vibrations of C–O–C at 1240 cm^−1^ and 1189 cm^−1^, and C–O stretching vibration at 1108 cm^−1^ [44]. The characteristic absorption peaks of PEO and PCL at 841 and 734 cm^−1^, respectively, contribute to the crystalline phase of the copolymers, suggesting the presence of some PCL and PEO crystalline phases in the copolymer micelles [45].

### 3.3. Physicochemical Characterization of the LSR-Loaded PEO-b-PCL Nanocarriers

**Figure 2:**
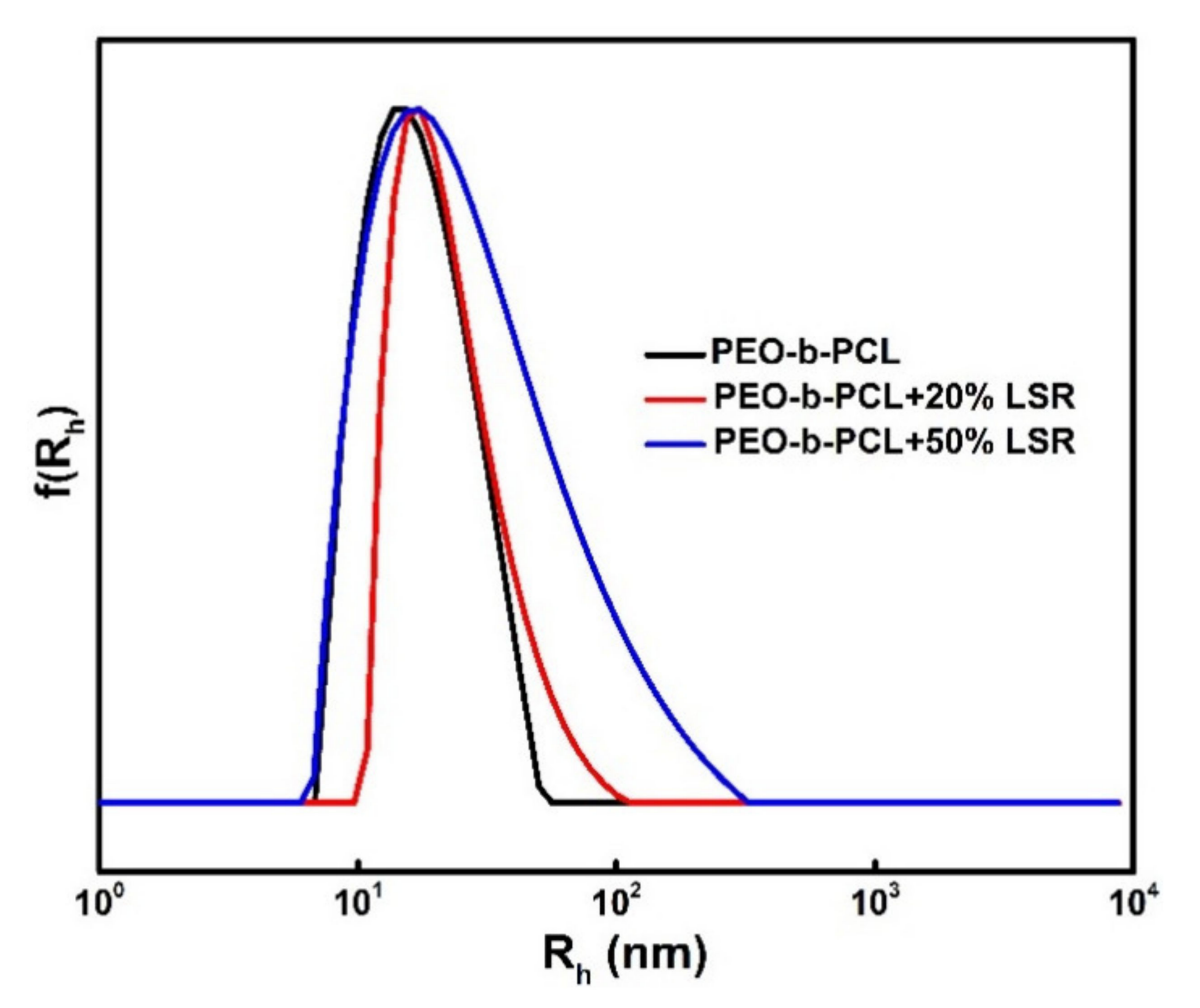
Comparison of the size distributions from Contin analysis for the PEO-b-PCL micelles, PEO-b-PCL+20%LSR, and PEO-b-PCL+50%LSR nanocarriers at 90° in aqueous solutions using the organic solvent evaporation method.

DLS, ELS, ATR-FTIR, and UV-Vis studies were carried out to assess the size, the surface potential and to examine drug encapsulation into the polymeric micellar core of the LSR-loaded PEO-b-PCL nanocarriers. The measurements were performed at 10^−3^ g/mL copolymer concentration, pH = 7, and at 25 °C. A comparison of size distributions of the PEO-b-PCL micelles and LSR-loaded PEO-b-PCL nanocarriers prepared by OSEM is provided in Figure 2. Broader size distributions are present in the cases of PEO-b-PCL+20% LSR (red line) and PEO-b-PCL+50% LSR (blue line) nanocarriers, compared to neat PEO-b-PCL micelles (black line), with a noticeable shoulder appearing in both cases at higher values of R_h_, potentially revealing the existence of additional species or the absence of uniform morphologies. DLS and SLS results highlight a significant increase in size (R_h_) and mass (based on determined scattered intensity from the solutions studied) of the LSR-loaded nanocarriers in both cases, implying a successful encapsulation of hydrophobic LSR into the polymeric micelles and some detectable changes in the structural characteristics of the drug-loaded copolymer micelles in comparison to empty ones. 

ELS measurements display strongly negative ζ_pot_ values (Table 2) for the LSR-loaded PEO-b-PCL nanocarriers, compared to PEO-b-PCL micelles, as encapsulated LSR carries one net negative charge, and this may contribute to the increase in the observed surface charge of the drug-loaded micelles [46]. The R_h_, PDI, scattered light intensity, and ζ_pot_ values of the LSR-loaded PEO-b-PCL nanocarriers are provided in Table 2. Apparently, LSR encapsulation results in an increase in the size polydispersity of the micellar carriers probably because of the expected increase in the hydrophobic content of the mixed nanocarriers.

**Figure 3:**
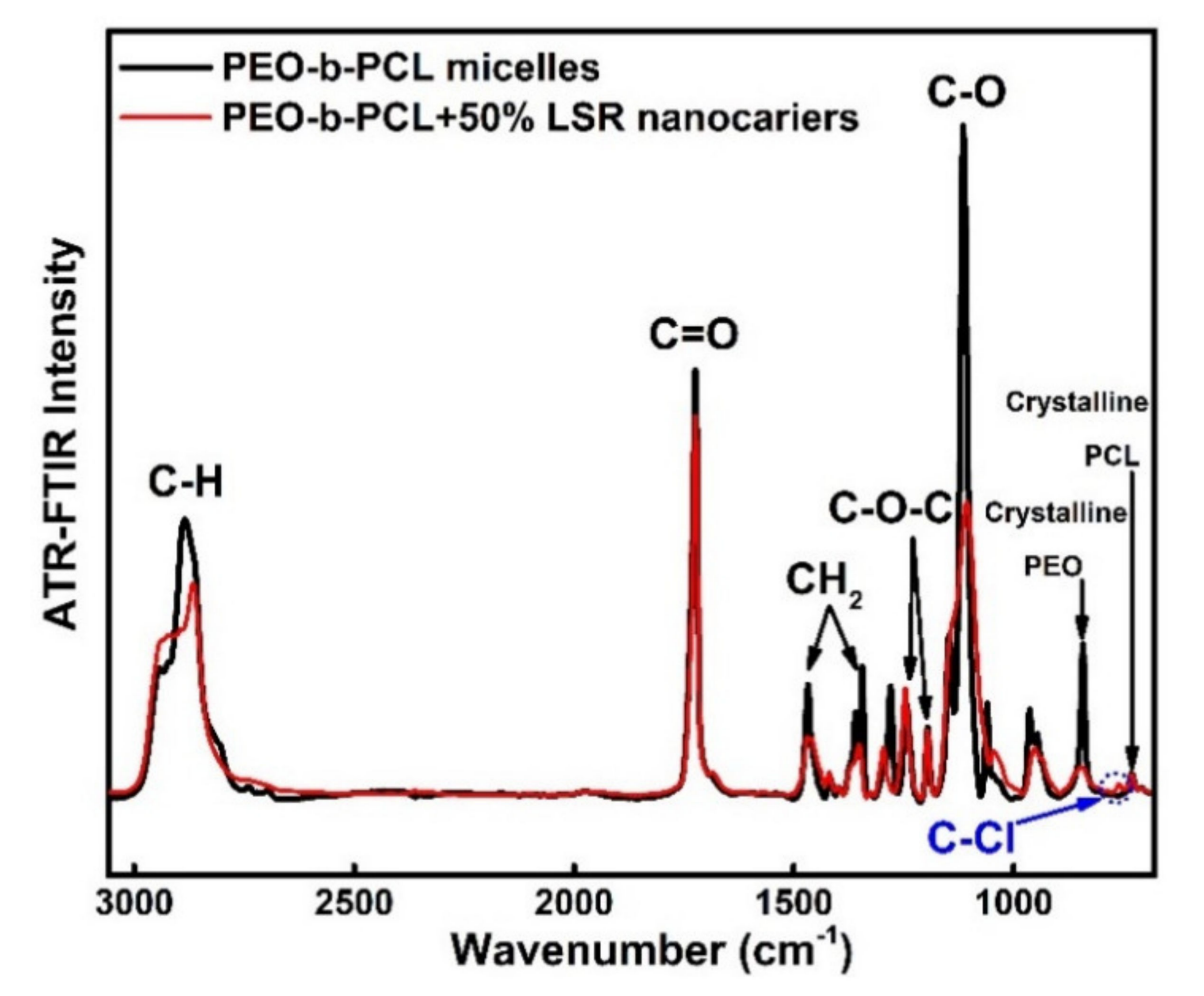
Comparison of ATR-FTIR spectra for PEO-b-PCL micelles and PEO-b-PCL+50%LSR nanocarriers in aqueous solutions using the organic solvent evaporation method.

ATR-FTIR and UV-Vis measurements were performed to examine LSR encapsulation into the polymeric core of the PEO-b-PCL micelles. A comparison of the ATR-FTIR spectra of the PEO-b-PCL micelles and PEO-b-PCL+50% LSR nanocarriers is provided in Figure 3. A significant decrease in ATR-FTIR intensity peaks is noticed after the 50% LSR encapsulation (red line), implying structural changes in the mixed copolymer/drug system. The appearance of new characteristic peaks is hardly observed. However, the appearance of new characteristic absorption peak at 762 cm^−1^ (blue annotation) may be attributed to C-Cl stretching modes [47], confirming the existence of LSR in the mixed copolymer/drug aqueous solution. 

**Figure 4:**
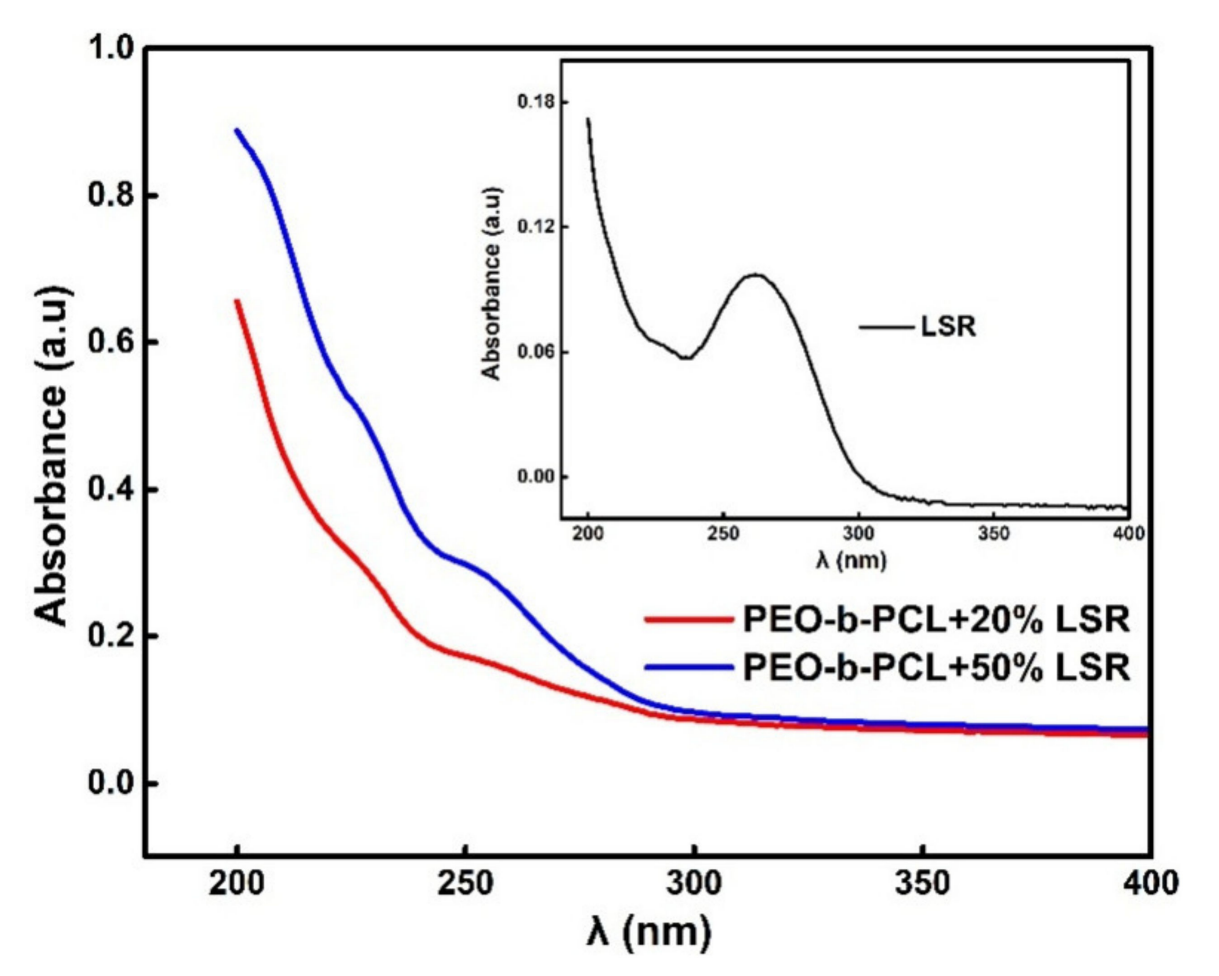
Comparison of UV-Vis spectra for LSR-loaded PEO-b-PCL nanocarriers.

UV-Vis spectroscopy studies further confirm the successful encapsulation of LSR into the PEO-b-PCL polymeric micelles. Figure 4 presents the UV-Vis spectra of PEO-b-PCL+20% LSR (red line) and PEO-b-PCL+50% LSR (blue line) nanocarriers in aqueous solutions along with the corresponding spectra of LSR (black line) in acetone. According to the literature, UV spectrum of LSR exhibits maximum absorbance at 205 nm, 225 nm, and 254 nm wavelength [48,49,50,51]. However, the UV-Vis spectra of LSR depicted in Figure 4 display a characteristic absorption peak at 260 nm and a lower absorbance at 228 nm. Knowing that PEO-b-PCL copolymer does not absorb at the respective wavelength range in the UV spectrum, the presence of the two UV absorption peaks (254 nm and 228 nm) of PEO-b-PCL+20% LSR and PEO-b-PCL+50% LSR nanocarriers in Figure 4 is related to the LSR UV absorption peaks verifying the successful drug loading into the hydrophobic core of the PEO-b-PCL micelles. The observed small shifts in the peak maxima may be correlated with copolymer/drug interactions (mainly hydrophobic and hydrogen bonding interactions).

### 3.4. Stability Studies of LSR-Loaded PEO-b-PCL Nanocarriers

**Figure 5:**
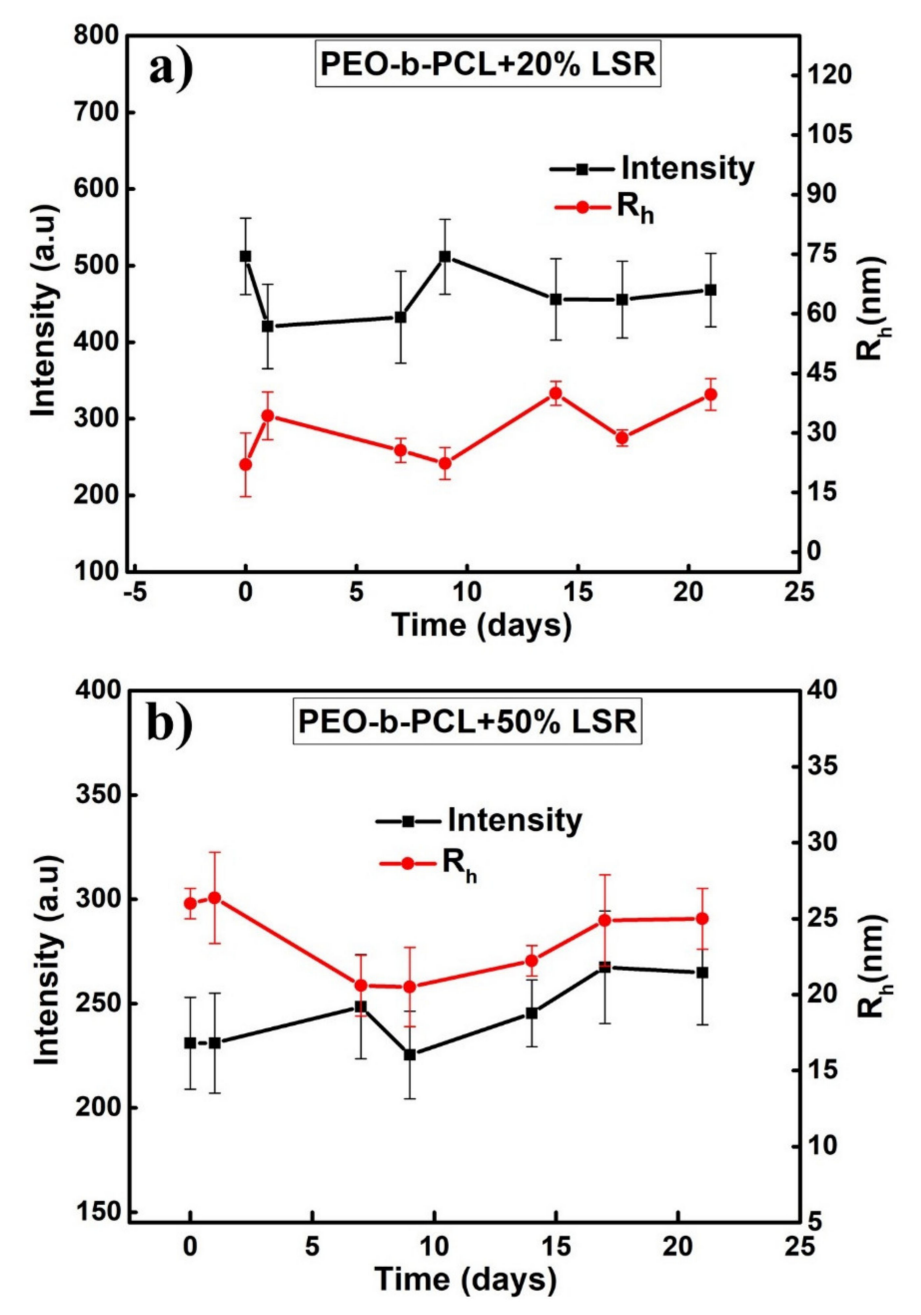
Scattered intensity and R_h_ measurements versus time obtained from DLS of (**a**) PEO-b-PCL+20% LSR and (**b**) PEO-b-PCL+50% LSR nanocarriers in aqueous solutions.

DLS measurements were repeated to study the stability of the resulting LSR-loaded PEO-b-PCL micellar solutions in a period of 21 days. The scattered intensity and R_h_ measurements versus time presented in Figure 5 are the average of three measurements at 90 degrees over a period of 21 days. for the a) PEO-b-PCL+20% LSR and b) PEO-b-PCL+50% LSR nanocarriers. The drug solutions exhibited prolonged stability (3 weeks). Specifically, the PEO-b-PCL+20% LSR nanocarriers do not exhibit significant intensity fluctuations, leading to shrink-resistant mixed nanostructures, while R_h_ substantially changes over time, highlighting an increase in the size of the mixed nanostructures from 22 nm (0 days) to 40 nm (21 days). Slight variations in scattered intensity and R_h_ are present in the case of PEO-b-PCL+50% LSR nanocarriers, suggesting a highly stable drug–polymer system within 21 days.

### 3.5. ^1^H-NMR Studies on PEO-b-PCL Micelles and LSR-Loaded PEO-b-PCL Nanocarriers

**Figure 6:**
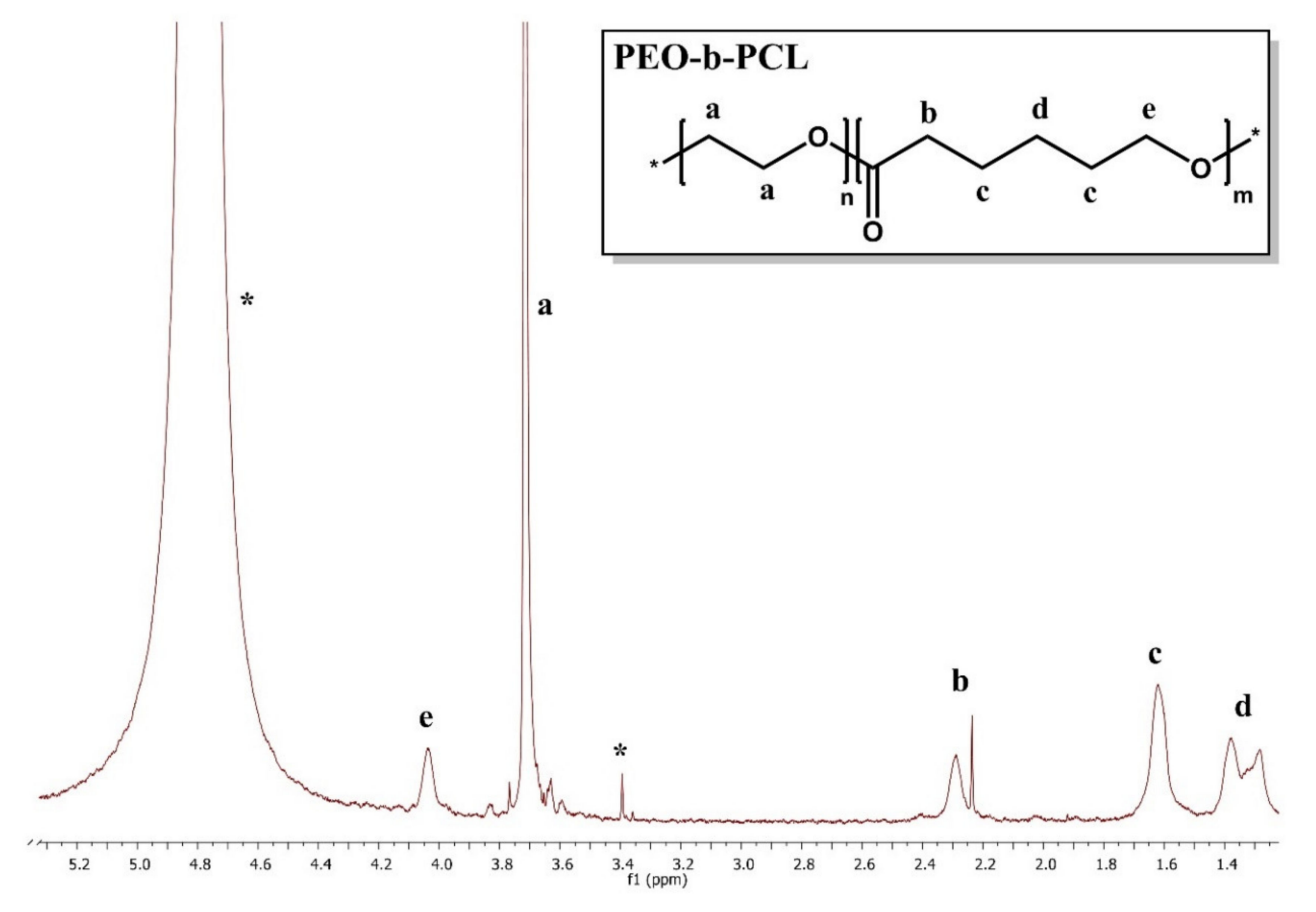
^1^H-NMR spectrum for the PEO-b-PCL micelles in D_2_O solutions, where the black letters denote the protons of the copolymer structure (a–e) and the asterisk refers to the solvent D_2_O.

**Figure 7:**
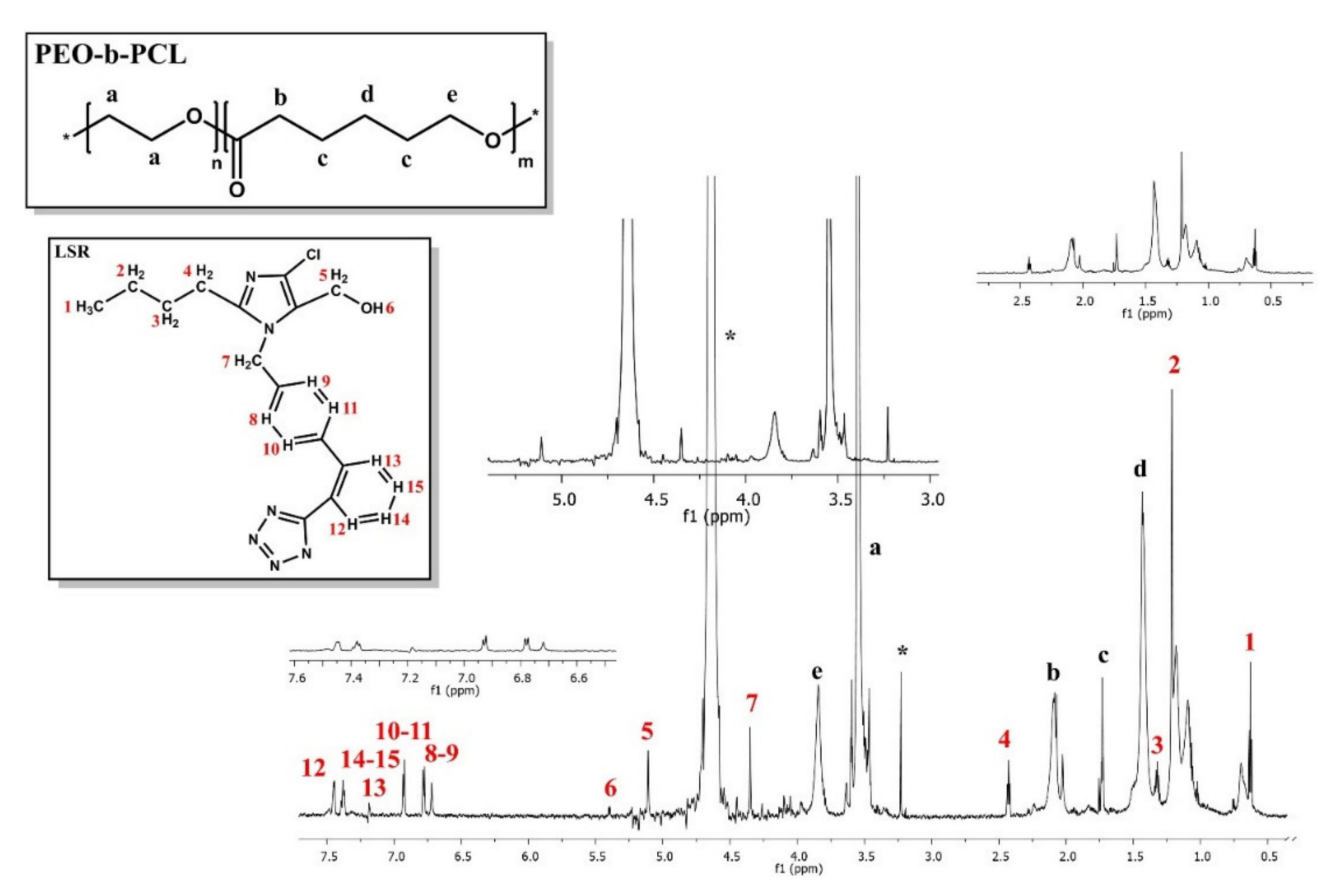
**^1^**H-NMR spectrum for the PEO-b-PCL+50% LSR nanocarriers in D_2_O solutions, where the black letters denote the protons of the copolymer structure (a–e), the red letters point to the LSR peaks and the asterisk refers to the solvent D_2_O.

^1^H-NMR studies were conducted on PEO-b-PCL micelles and PEO-b-PCL+50% LSR nanocarriers, prepared by utilizing the OSEM in D_2_O solutions, in order to examine the aggregation behavior of the micelles, as well as to verify the presence of LSR in the polymer–drug solutions. Structural elucidation of PEO-b-PCL diblock and LSR have been reported in previous publications [51,52,53,54]. The ^1^H-NMR spectra for the PEO-b-PCL micelles and PEO-b-PCL+50% LSR nanocarriers are observed in Figure 6 and Figure 7. The black letters in the ^1^H-NMR spectra denote the protons of the copolymer structure, whereas the red letters depicted in the ^1^H-NMR spectrum of PEO-b-PCL+50% LSR nanocarriers point to the LSR peaks. As noticed in Figure 7, the proton signals of LSR are clearly evident in the ^1^H-NMR spectrum of the PEO-b-PCL+50% LSR nanocarriers, confirming the encapsulation of LSR in the polymeric nanocarriers.

A comparison of the observed peaks of losartan with those reported in organic solvents and micelles signifies its high flexibility. The pattern of H12-H15 is simplified signifying the free rotation of the not-bridged aromatic ring. The chemical shifts of the PEO-b-PCL proton signals in the absence (Figure 6) and in the presence of LSR (Figure 7) are gathered in Table 3. Furthermore, the chemical shifts of LSR proton signals in the presence both of PEO-b-PCL copolymer and sodium dodecyl sulfate (SDS) micelles [27] are displayed in Table 4.

### 3.6. 2D- COSY Studies on LSR-Loaded PEO-b-PCL Nanocarriers

**Figure 8:**
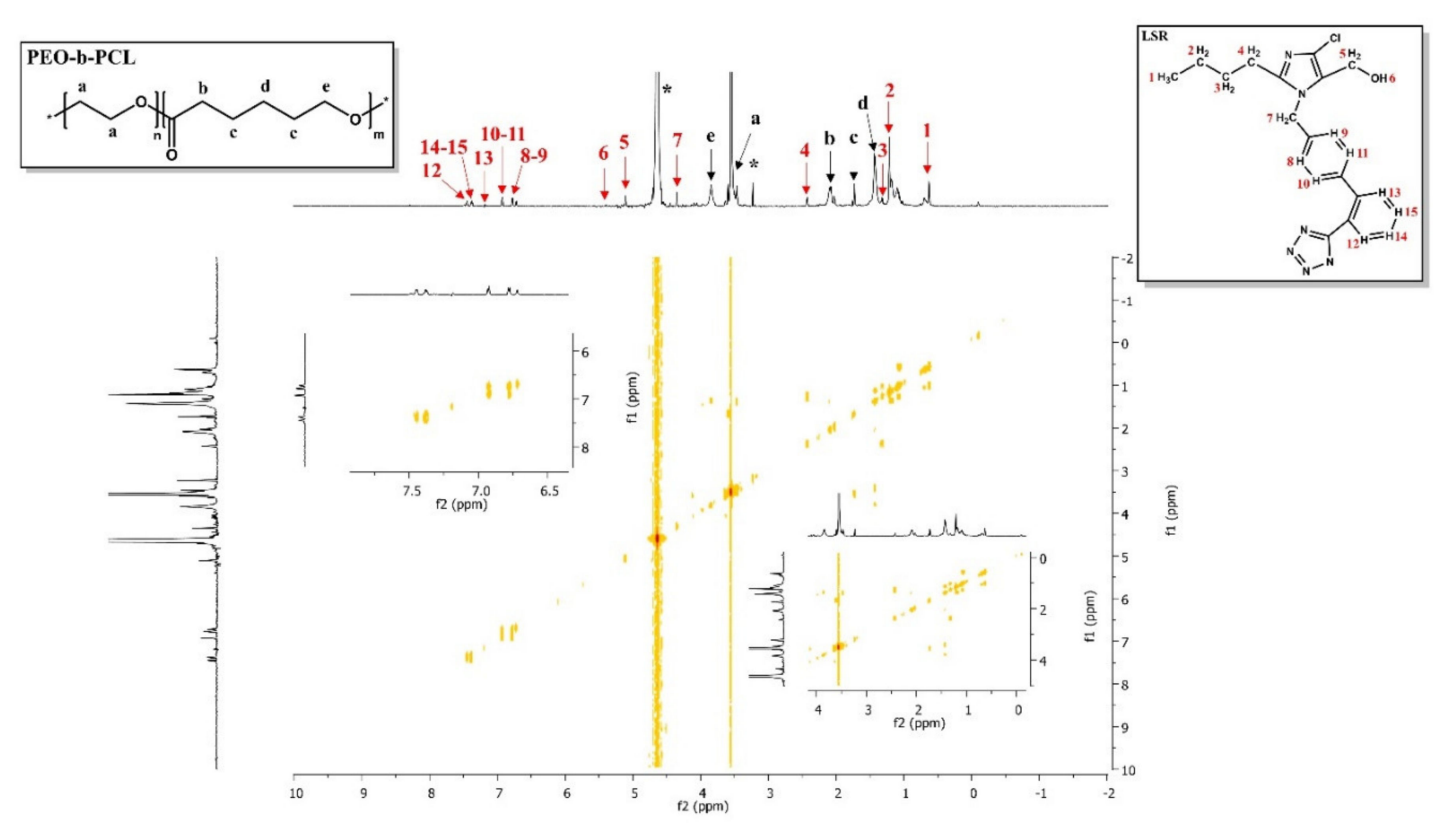
2D-COSY spectrum of PEO-b-PCL+50% LSR nanocarriers, where the black letters (a–e) denote the protons of the copolymer structure, the red letters point to the LSR peaks and the asterisk refers to the solvent D_2_O. Eminent correlations are those between the protons within the copolymer and LSR.

2D-COSY experiments on PEO-b-PCL+50% LSR nanocarriers were conducted to confirm the internal structure and detect the intramolecular interactions between segments of the PEO-b-PCL copolymer and LSR moieties. Figure 8 presents the 2D-COSY spectrum of the PEO-b-PCL+50% LSR nanocarriers in D_2_O where the black arrows denote the protons of the copolymer structure while the red ones point to the LSR peaks. The cross peaks observed between the protons of the copolymer and the protons of LSR in the 2D-COSY spectrum in Figure 8 are related to the intramolecular interactions, which further elucidate the structure of both copolymer and LSR. 

### 3.7. 2D-NOESY Studies on LSR-Loaded PEO-b-PCL Nanocarriers

**Figure 9:**
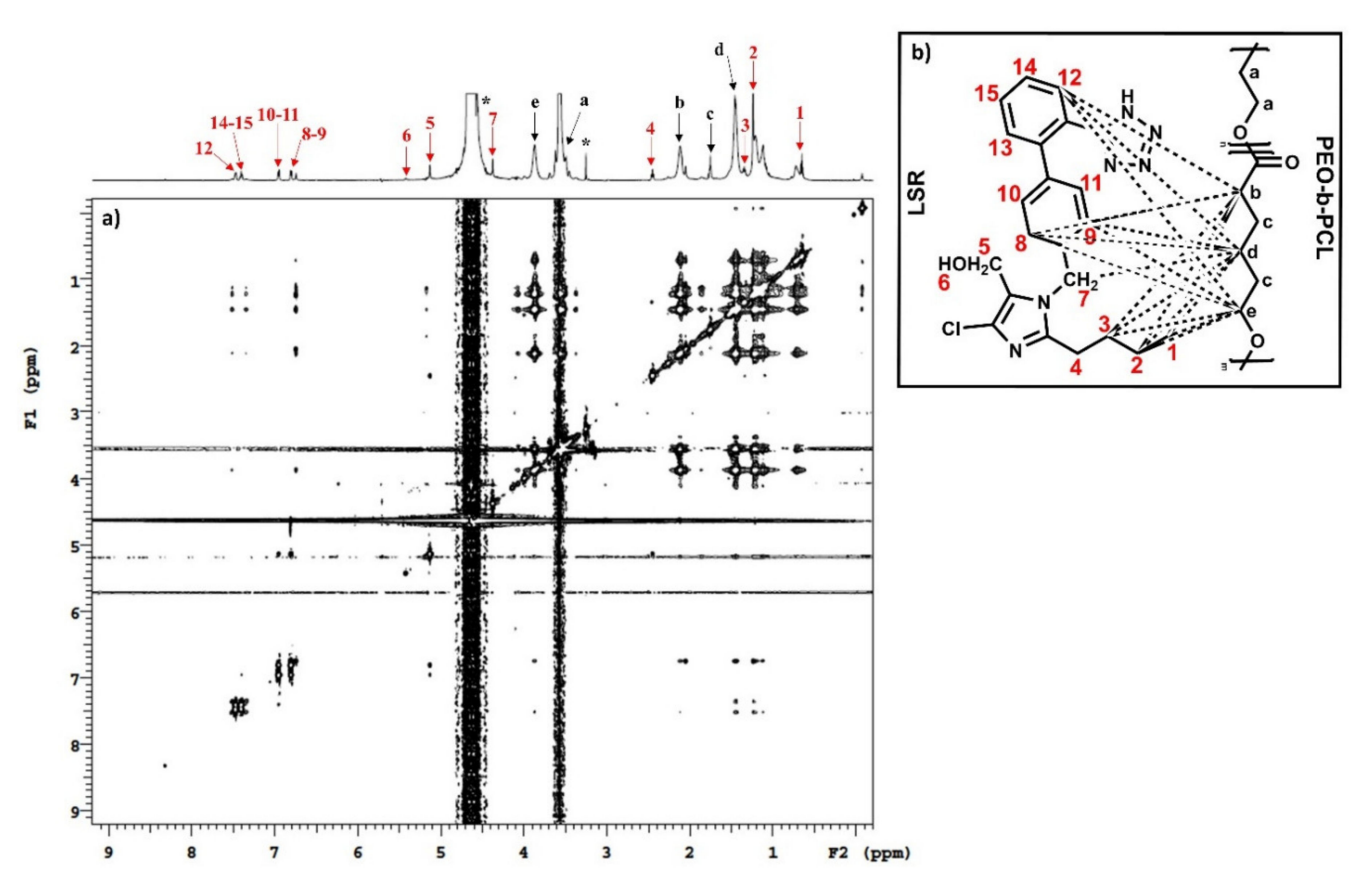
(**a**) 2D-NOESY spectrum of PEO-b-PCL+50% LSR nanocarriers and (**b**) schematic approach of LSR in PEO-b-PCL micelles. The black letters (a–e) denote the protons of the copolymer structure, the red letters point to the LSR peaks and the asterisk refers to the solvent D_2_O.

2D-NOESY experiments on PEO-b-PCL+50% LSR nanocarriers were performed to identify the interacting groups and to investigate the intra- and intermolecular interactions between the PEO-b-PCL diblock copolymer and LSR, and lastly, to corroborate the successful encapsulation of LSR into the polymeric PEO-b-PCL micelles. Evidence for the intermolecular association was obtained from the 2D-NOESY spectrum of PEO-b-PCL+50% LSR nanocarriers (Figure 9) in D_2_O, where the black arrows denote the protons of the copolymer structure and the red arrows point to the LSR peaks. Figure 9 shows cross peaks between the phenyl rings (8–9, 12) and butyl chain of LSR (1, 2, 3) with the methylene signals of PCL (b, d, e). According to the most intense NOEs, LSR must approach PEO-b-PCL micelles, as it is indicated in the Figure 9. Particularly, the biphenyl ring (8–9, 12) and the butyl chain (1, 2 and 3) of LSR approach the methylene signals of PCL in positions b-d-e, indicating that the hydrophobic interactions are mainly exerted by the biphenyl ring and the butyl chain, respectively.

### 3.8. 2D-DOSY Studies on LSR-Loaded PEO-b-PCL Nanocarriers

**Figure 10:**
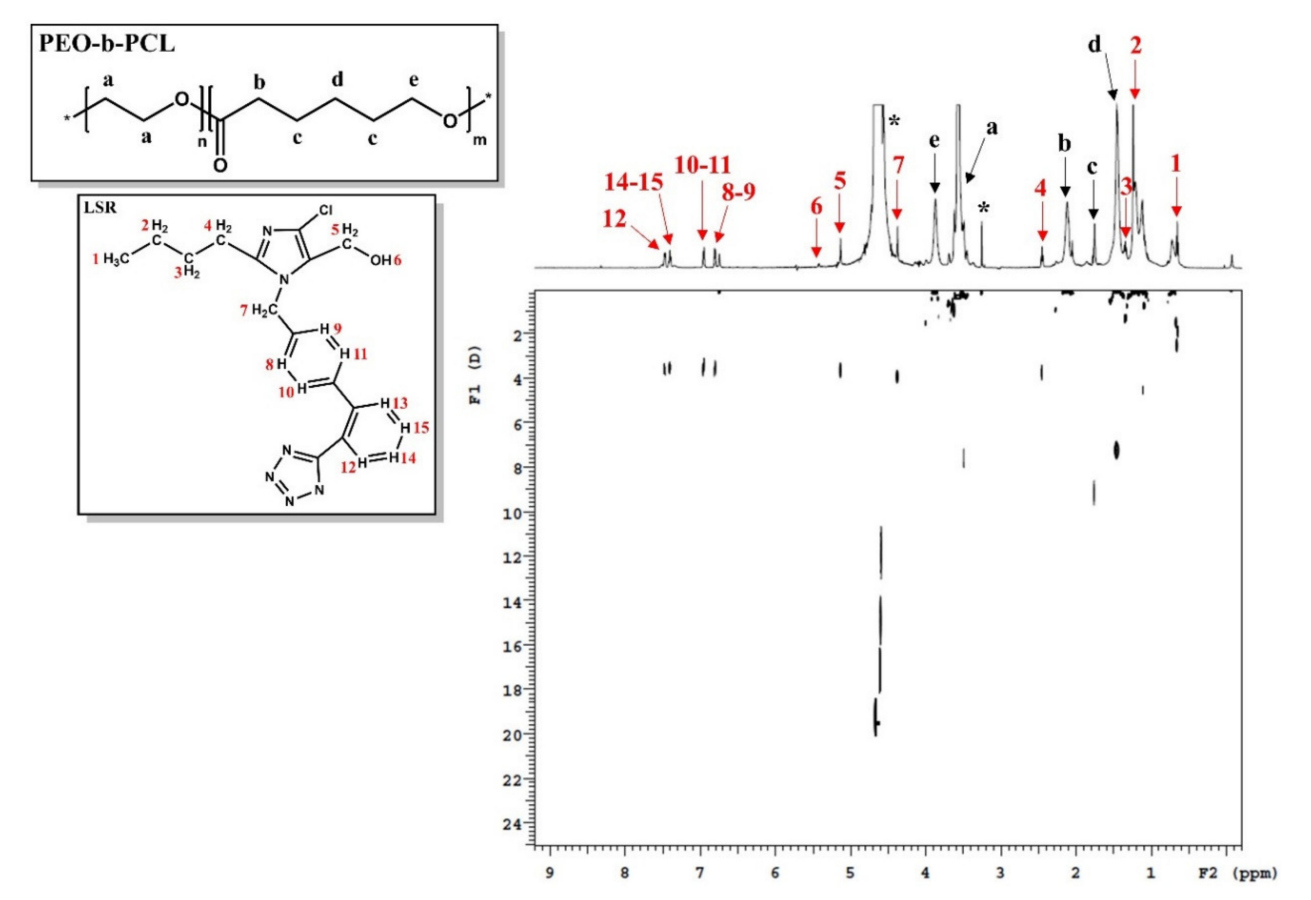
2D-DOSY spectrum of PEO-b-PCL+50% LSR nanocarriers. The black letters (a–e) denote the protons of the copolymer structure, the red letters point to the LSR peaks and the asterisk refers to the solvent D_2_O.

The PEO-b-PCL+50% LSR nanocarriers were further characterized using 2D-DOSY experiments to detect the presence of assemblies, to probe drug association with micelles, and to determine the self-diffusion coefficients D. Diffusion experiments displayed in Figure 10 confirmed the association of LSR with PEO-b-PCL micelles. The black arrows depicted in Figure 10 denote the protons of the copolymer structure and the red arrows point to the LSR peaks. Specifically, a double series of traces with low constants (D = 0.14 × 10^−10^ m^2^ s^−1^ and D = 3.57 × 10^−10^ m^2^ s^−1^) is evident, implying a relatively slow chemical exchange between the free and the micelle-bound states of the drug. 

### 3.9. ^1^H-NMR Temperature Studies on PEO Homopolymer and PEO-b-PCL Copolymer

A systematic ^1^H-NMR study for the PEO-b-PCL copolymers over a wide range of temperatures to trace the mobility of protons during micellization is still very scarce. 

**Figure 11:**
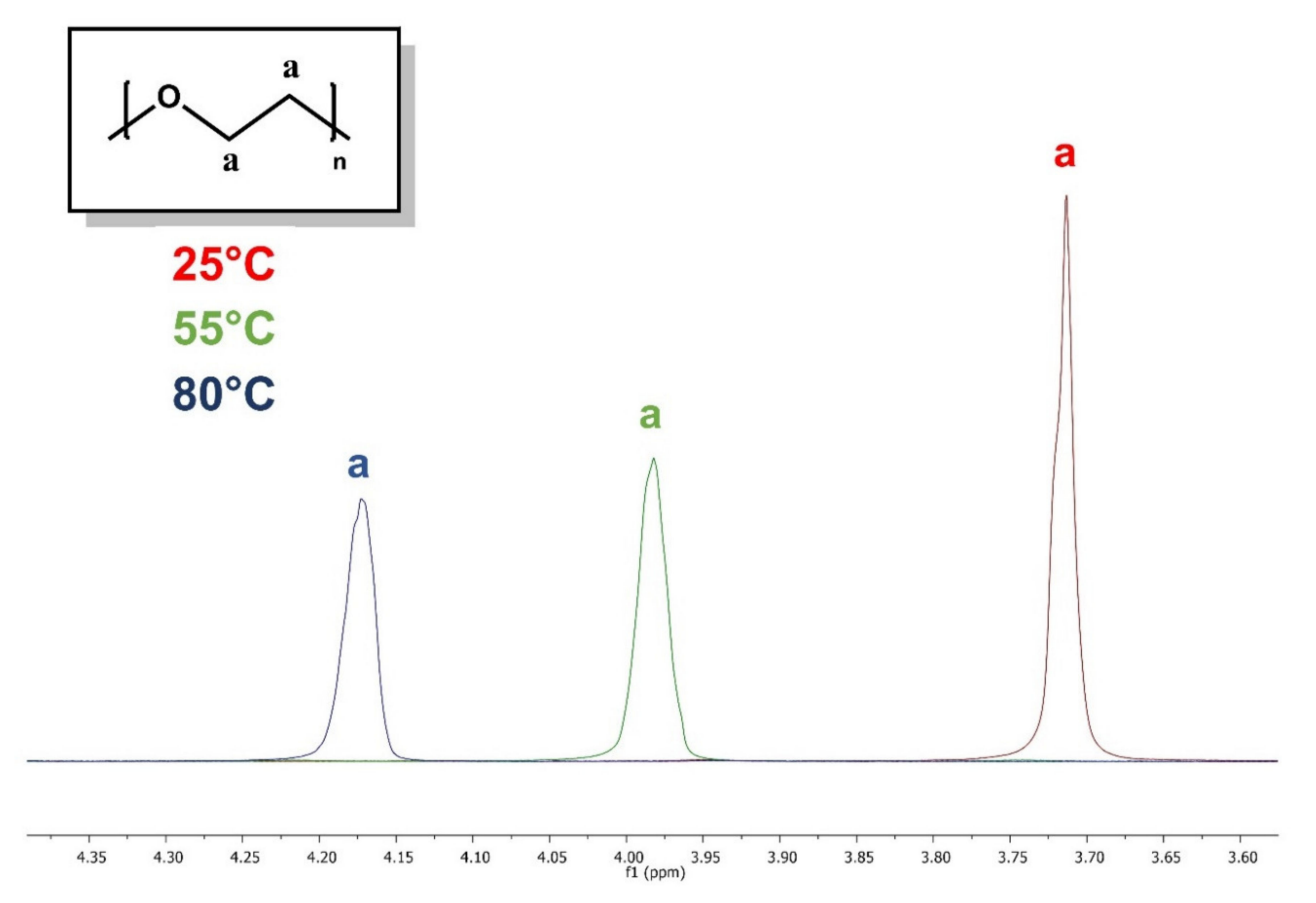
Comparison of ^1^H-NMR spectra of PEO homopolymer at various temperatures, where the red, green, and blue letter (a) refers to the protons of PEO homopolymer at 25 °C, 55 °C and 80 °C, respectively.

**Figure 12:**
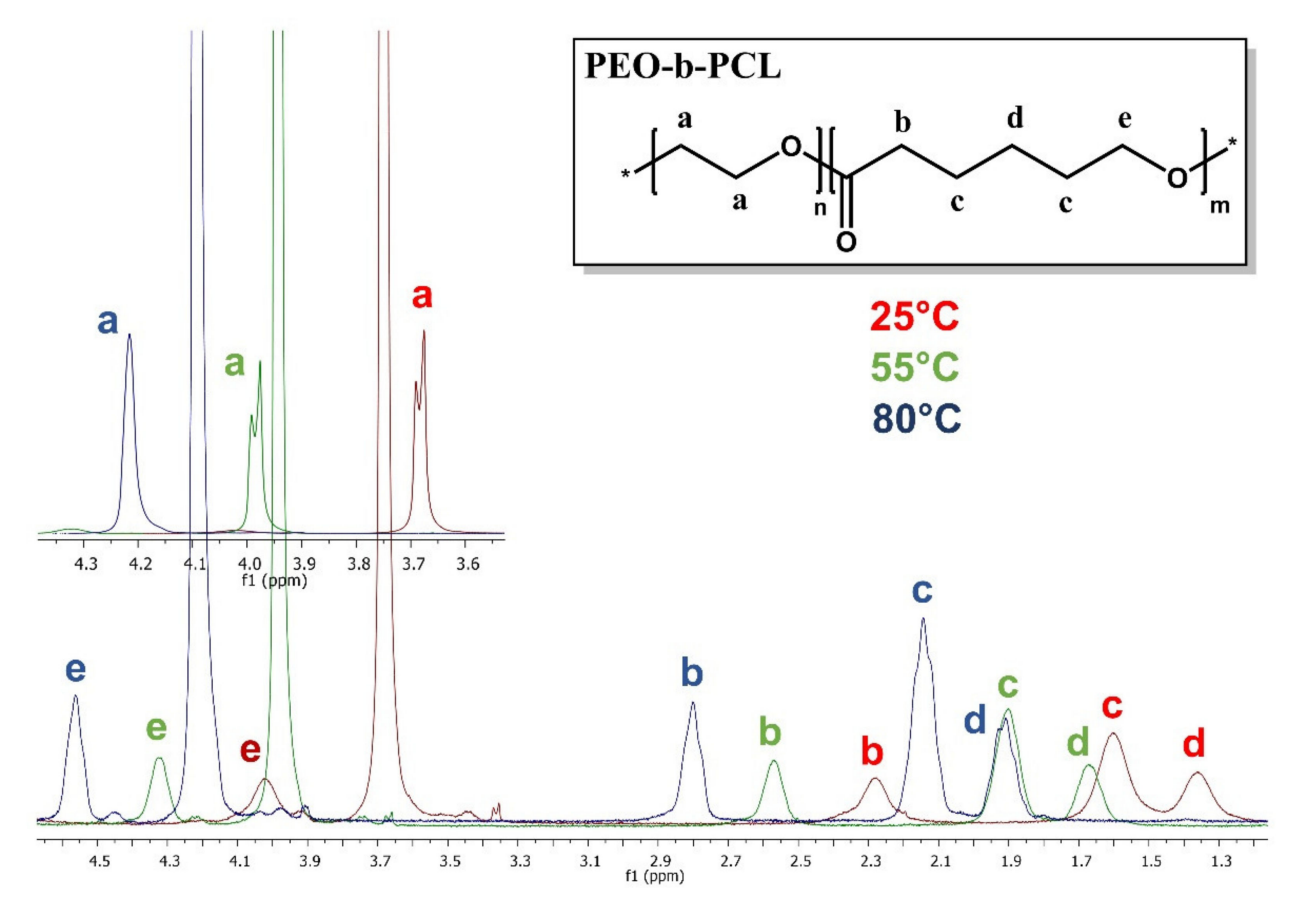
Comparison of ^1^H-NMR spectra of -PEO-b-PCL diblock at various temperatures. The red, green, and blue letters refer to the protons of PEO-b-PCL copolymer at 25 °C, 55 °C and 80 °C, respectively. The black letters (a–e) denote the protons of the copolymer structure. Asterisk in the chemical structure of the copolymer denotes the presence of end groups at the ends of the copolymer chain.

The ^1^H-NMR spectra of PEO homopolymer and PEO-b-PCL diblock at three different temperatures are juxtaposed in Figure 11 and Figure 12. The red, green, and blue letters depicted in both ^1^H-NMR spectra denote the protons of the copolymer structure at 25 °C, 55 °C and 80 °C, respectively.

The ^1^H-NMR spectrum in Figure 11 demonstrates a decrease in PEO’s proton mobility with increasing temperature. Specifically, the half-width is increased, while the integrated intensity of the proton signals is decreased with increasing temperature. The water solubility of PEO derives from the competition between PEO–water and water–water hydrogen bonding, delicately balanced by hydrophobic interactions elicited by the ethylene groups. The rupture of hydrogen bonds with increasing temperature is responsible for the decrease in its solubility upon heating [55]. 

Interestingly, the ^1^H-NMR peaks of PEO-b-PCL diblock in Figure 12 are shifting with increasing temperature. At elevated temperatures (55 °C and 80 °C), the proton signals are becoming narrower and more intense, indicating enhanced mobility of the protons. 

The proton mobility of PEO still remains limited in the presence of PCL, leading to lower-intensity proton signals at temperatures greater than 25 °C.

According to the literature, PEO-b-PCL is a temperature-independent amphiphilic copolymer, which contains a semi-crystalline PCL hydrophobic and biodegradable polyester block. Moreover, PCL has a low melting point of around 60 °C and a glass transition temperature (T_g_) of about −60 °C [44]. As a result, a temperature increase may elicit water penetration of the PCL core, causing partial hydrolysis throughout the entire polymer core matrix and leading to enhanced proton mobility.

## 4. Conclusions

Highly stable drug-loaded biocompatible/biodegradable nanocarriers formed by encapsulation of LSR into an amphiphilic PEO-b-PCL diblock copolymer were developed and studied using a variety of physicochemical techniques.

The PEO-b-PCL diblock copolymer formed micelles of R_h_ = 17 nm in aqueous milieu, while the PEO-b-PCL+20% LSR (R_h_ = 22 nm) and PEO-b-PCL+50% LSR (R_h_ = 26 nm) nanocarriers highlighted a significant increase in their size and mass based on DLS data. The PEO-b-PCL micelles and LSR-loaded PEO-b-PCL nanocarriers exhibited slightly negative ζ_pot_ values. The LSR-loaded PEO-b-PCL nanocarriers proved to be highly stable during a period of 21 days according to DLS stability studies. ATR-FTIR measurements verified the chemical structure of PEO-b-PCL diblock and a new LSR-related absorption peak appeared at the ATR-FTIR spectrum of the LSR-loaded PEO-b-PCL nanocarriers.

^1^H-NMR measurements verified the existence of LSR into PEO-b-PCL micelles, while 2D-COSY, 2D-NOESY, and 2D-DOSY experiments confirmed the internal structure of PEO-b-PCL copolymer and LSR moieties and further proved the successful encapsulation of LSR into the PEO-b-PCL micelles. Particularly, 2D-NOESY experiments evidenced the intermolecular association between the biphenyl ring and butyl chain of LSR structure with the methylene signals of PCL.

Noteworthily, ^1^H-NMR studies as a function of temperature showed signs of decreased proton mobility on PEO homopolymer due to the rupture of hydrogen bonds with increasing temperature. On the other hand, the PEO-b-PCL micelles exhibited enhanced proton mobility, probably caused by the melting of the PCL hydrophobic core upon increasing temperature. 

The physicochemical results of the present LSR-loaded PEO-b-PCL nanocarriers set the stage for further biopharmaceutical evaluation and imaging characterization to promote their candidacy as nano-drug delivery systems.

## Figures and Tables

**Table 1:** Molecular characteristics of the PEO-b-PCL copolymer.

Sample	Mn ^a^ Polymer (g/mol)	Mn ^b^ PEO (g/mol)	M_w_/M_n_^a^	% PCL ^c^	M_w,true_^c^
PEO-b-PCL	7500	5000	1.18	30	7100

^(**a**)^ Determined by size-exclusion chromatography (SEC) in THF/3%v Et_3_N. ^(**b**)^ PEO 5000 was purchased and used as macroinitiator. ^(**c**)^ Calculated from ^1^H-NMR spectrum.

**Table 2:** Dynamic and electrophoretic light scattering (DLS and ELS) results for the PEO-b-PCL copolymer and LSR-loaded PEO-b-PCL nanocarriers in aqueous solutions. PDI = polydispersity index.

Sample	R_h_ (nm)	PDI	Intensity (a.u)	ζ_pot_ (mV)
PEO-b-PCL	17	0.183	92	−6
PEO-b-PCL+20% LSR	22	0.321	512	−21
PEO-b-PCL+50% LSR	26	0.46	231	−17

**Table 3:** H-NMR chemical shifts of PEO-b-PCL copolymer: (a) in the absence of LSR and (b) in the presence of LSR in D_2_O solutions.

	^1^H-NMR Chemical Shifts (ppm)	
Protons of the Copolymer Structure	Proton Signals of PEO-b-PCL in the Absence of LSR	Proton Signals of PEO-b-PCL in the Presence of LSR
Ha	3.72	3.56
Hb	2.23	2.08
Hc	1.62	1.73
Hd	1.33	1.44
He	4.03	3.84
*Reference*	D_2_O: 4.73	

**Table 4:** H-NMR chemical shifts of LSR: (a) in the presence of PEO-b-PCL copolymer and (b) in the presence of sodium dodecyl sulfate (SDS) micelles in D_2_O solutions.

	^1^H-NMR Chemical Shifts (ppm)	
Protons of LSR Structure	Proton Signals of LSR in PEO-b-PCL	Proton Signals of LSR in SDS Micelles
H1	0.63	0.77
H2	1.22	1.22
H3	1.32	1.5
H4	2.43	2.52
H5	5.12	4.39
H6 (OH)	5.4	-
H7	4.35	5.15
H8- H9	6.78	6.85
H10- H11	6.93	6.99
H12	7.45	7.64
H13	7.19	7.27
H14- H15	7.38	7.34–7.41
*Reference*	D_2_O: 4.73	
